# Previous reproductive success informs nest-building decisions

**DOI:** 10.1093/beheco/araf009

**Published:** 2025-02-06

**Authors:** Evelyn J Alexander, Sophie C Edwards, Elisabeth G Chapman, Susan D Healy

**Affiliations:** Centre for Biological Diversity, School of Biology, Sir Harold Mitchell Building, University of St Andrews, St Andrews, Fife, KY16 9TH, United Kingdom; The Lyell Centre, Institute for Life and Earth Sciences, Research Avenue South, Heriot-Watt University, Edinburgh, EH14 4AP, United Kingdom; Centre for Biological Diversity, School of Biology, Sir Harold Mitchell Building, University of St Andrews, St Andrews, Fife, KY16 9TH, United Kingdom; School of Psychology & Neuroscience, St Mary's Quad, South Street, University of St Andrews, Fife, KY16 9JP, United Kingdom; Centre for Biological Diversity, School of Biology, Sir Harold Mitchell Building, University of St Andrews, St Andrews, Fife, KY16 9TH, United Kingdom; Centre for Research in Animal Behaviour, Department of Psychology, Washington Singer Laboratories, University of Exeter, Exeter, EX4 4QG, United Kingdom; Centre for Biological Diversity, School of Biology, Sir Harold Mitchell Building, University of St Andrews, St Andrews, Fife, KY16 9TH, United Kingdom

**Keywords:** blue tit, nest building, nest design, reproductive success, reproductive experience

## Abstract

Behavioral outcomes, including foraging efficiency and reproductive success, often improve with age and with repeated breeding attempts. Here we examined the role of previous experience on the nest-building behavior of wild blue tits *Cyanistes caeruleus*. In particular, we focused on how previous success or failure in rearing nestlings shapes nest-building decisions in subsequent years. We found that previous breeding *outcome* is important for predicting nest building decisions in blue tits: birds that had previously raised fledglings added insulating material at a similar rate as they had when they built their first nest. Some birds that had been previously unsuccessful changed their rate of adding insulating material and then were more likely to produce fledglings. These responses indicate that at least some of the decisions made by wild nest-building birds are based on their own experience of past breeding seasons.

## Teaser

We investigated how past reproductive experience influenced the nest-building behavior of wild blue tits. Previously successful birds built their next nest the same way as they had their first, whereas previously unsuccessful birds did not. Some previously unsuccessful birds changed their nest-building strategy and were more likely to produce live young from their next nest. These observations support previous experiments that showed birds use their experience of past success when they build their subsequent nest.

## Introduction

Behavioral plasticity allows animals to respond to environmental variation, over the short or long term. Behavioral modifications can be innate, with variation in behavioral phenotypes occurring with variation in local environmental conditions, or may be learned, through one’s own experience or socially (Mery and Burns 2010). Learning new information has been linked to various measures of fitness, including improved fecundity and invasion success (Sol et al. 2002; Robertson and Olsen 2015).

Experience can modify a range of behaviors, including foraging, migration, and reproduction. For example, with experience some seabirds improve their foraging efficiency and the quality of food they provide to their offspring (Daunt et al. 2007; Limmer and Becker 2009), and as they aged, black kites *Milvus migrans* departed and arrived on their migration routes earlier (Sergio et al. 2014). Furthermore, female zebra finches *Taeniopygia guttata* and three-spined sticklebacks *Gasterosteus aculeatus* L. modify their choice of mate based on the perceived quality of previously encountered males (Bakker and Milinski 1991; Collins 1995), while white storks *Ciconia ciconia* are more likely to reuse a nest site from which they have previously successfully fledged young (Bialas et al. 2023).

Nests are built by a wide diversity of animals for incubating and protecting young, for shelter, and sometimes for courtship (eg by termites *Macrotermes subhyalinus Rambur* (Weir 1973), Siamese fighting fish *Betta splendens Regan* (Jaroensutasinee and Jaroensutansinee 2001), and gorillas *Gorilla gorilla* (Mehlman and Doran 2002)). But nest building is particularly widespread and varied in birds, making them useful for the investigation of both inter- and intra-specific variation in this behavior (Healy et al. 2023). Here we ask whether nest building decision-making correlates with the well-recorded pattern that birds, as they age and have increasing breeding experience, typically increase their success in rearing fledglings (eg Nol and Smith 1987; Reid et al. 2003; Nisbet and Dann 2009; Janiszewski et al. 2017).

The dependence of avian eggs on a narrow temperature range (approximately 36 °C to 40 °C) for the embryo to develop normally (DuRant et al. 2013) suggests that selection of appropriately insulating materials should be directly linked to reproductive success. This is because this temperature range is substantially above the ambient temperatures typically experienced by birds breeding in northern temperate regions: the temperature range at our field site during the breeding season (March to May) in the years studied was −3.8 to 24.5 °C, with mean of 8.2 °C. Evidence for the importance of nest insulation comes from, among others, tree swallows *Tachycineta bicolor* that had feathers experimentally removed from their nests. These birds fledged fewer offspring than did those birds with feathers remaining in their nests (Lombardo et al. 1995), while in blue tits *Cyanistes caeruleus*, the greater the mass of insulating lining material in the nest, the more likely the eggs are to hatch (Glądalski et al. 2016). However, the incubating parent bird, whose presence on the nest also influences the temperature experienced by embryos, can attempt to mitigate the effects of poorly insulated nests. For instance, village weavers *Ploceus cucullatus* with less-well insulated nests spent more time sitting on their eggs. But because time spent incubating is time that they cannot spend foraging, this compensation for below-par nest insulation has consequences for the parent. Therefore, a well-insulated nest may represent a trade-off made by the nest-building bird to optimize foraging and incubating time (White and Kinney 1974), particularly when air temperatures are cool (eg springtime at high latitudes).

There is increasing evidence that experience of building can affect choices made by nest builders, at least in the laboratory. In particular, birds that have had success with their first breeding attempt tend to stick with the same material when they build their next nest, while birds that have been unsuccessful change their choice of materials or change how much material to use (Muth and Healy 2011; Edwards et al. 2020; Camacho-Alpízar et al. 2021). Birds building in the laboratory will also vary the materials they use depending on the structural properties of the materials offered (Bailey et al. 2014), possibly due to the costs of building with different materials (Breen et al. 2021). All these data, however, have come from the laboratory where birds build under relatively invariant temperatures and from a single species (zebra finches), demonstrating only what these birds *can* do (Pritchard et al. 2016). To determine what birds *actually* do, here we examine whether experience plays a similar role in decision-making in the wild, for birds that must choose among a variety of materials and are exposed to inter- and intra-year temperature variation.

Blue tits are a useful species for addressing this question as individuals tend to build their nests in the same area in successive years (Perrins 1979). For example, females in a Mediterranean population typically do not disperse between breeding seasons or move less than 100 m (García-Navas and Sanz 2011). The nest (built solely by the female) typically has a structural base consisting of moss and grass, and is lined with insulating material such as feathers and mammal hair (Mainwaring et al. 2012). Nests can only be deconstructed to examine material composition once, so instead we used a photographic sequence of the nest surface to investigate decision-making during nest building.

We examined whether reproductive success in blue tits affected their nest building behavior in three ways. Firstly, if a well-insulated nest is associated with improved breeding success in this population of blue tits, then we expected that birds that fledge more young would include insulation material into their nest earlier and/or faster than would birds that fail to fledge young. Secondly, based on laboratory data, we expected previously successful birds to base their building decisions on their own experience, repeating the rate and timing at which they added insulation material from their previous year. Conversely, we expected that previously unsuccessful birds were more likely to *change* the timing or rate at which they added insulating material into their next nest. Finally, we investigated whether the ambient temperature experienced during nest building influenced these choices. Air temperature at our northern hemisphere field site tends to increase as spring progresses, and blue tits add insulation material to their nests in preparation for egg laying and incubation. Therefore, within a breeding season (hereafter called year), *warmer* temperatures (typically occurring later during nest building) may act as a cue for the building female to increase the rate at which she adds insulation. We therefore expected to see the nest surface increasingly covered by insulation material (and therefore decreasing coverage by structural material) as the nest got nearer to the point at which the female laid her first egg. Furthermore, if first-time and previously unsuccessful birds pay more attention to temperature (rather than their own experience) when making decisions about nest materials than do previously successful birds (Edwards et al. 2020), we expected those birds to be especially responsive to warmer temperatures.

## Methods

### Field observations

We analyzed data collected from 204 nests built by 117 blue tits during five reproductive seasons, from 2017 to 2022. The nests were built in approximately 88 nestboxes (woodcrete Schwegler Nestbox 1B, with an internal cylindrical chamber 120 mm in diameter, and 170 mm wide by 180 mm deep), attached to trees in St Andrews, UK, at a variety of heights (1 m—2.5 m from the ground) and orientations. We inspected the nestboxes each year prior to the start of the blue tit breeding season, to ensure that they were all empty and clean. As data collection was not permitted in 2020 during the Covid-19 lockdown the following analyses do not include 2020 data.

In each year, nestboxes were checked every 4 d from March 1^st^ until initial nest material (moss or grass) was found in at least one box, from which point the frequency of checks was increased to every 2 d. At each check, the presence or absence of nest material, eggs, an incubating adult, and nestlings were recorded. If present, eggs, and later nestlings, were counted. Once incubation was recorded (by the presence of an adult sitting on four or more eggs, or the eggs were warm to the touch), check frequency was reduced to every 4 d. During incubation, binoculars were used to identify the adults by their color rings as they entered or left the nest. If we had not color-ringed them in a previous year of the study (due to previous reproductive attempts in a natural nest hole, or because this was their first reproductive attempt), adults were caught on the nest by blocking the entrance hole (Schlicht and Kempenaers 2015), sexed (by presence or absence of brood patch or cloacal protrusion), aged (1 yr or older (Svensson 1992)), color- and BTO-ringed (license number C6551). Nest checks continued every 4 d until fledging, after which we counted dead nestlings in the nestbox. Any nestlings not found dead were assumed to have fledged successfully.

We calculated nest building duration individually for each nest, as the number of days from the first observation of nest material to the day the first egg was laid. If more than one egg was present at the first observation to record eggs, we assumed eggs were laid at the rate of one per day (Perrins 1979).

At each check, a photograph of the upper nest surface was taken using an iPhone 5C, SE, 6 or 6S. To ensure as much of the nest surface as possible was visible in the image, a wide-angle lens was attached to the front-facing phone camera and the phone held under the ceiling of the nestbox to take the photo.

Hourly temperature data were obtained from the nearest Met Office weather station, Leuchars. Because Leuchars is only five miles north of St Andrews and due to the strong spatial autocorrelation observed in temperature data, we assumed the temperatures at the weather station were similar to those in St Andrews (Lennon and Turner 1995). From those data, we calculated daily mean summary temperature statistics.

### Ethical note

This study was approved by the University of St Andrews Animal Welfare and Ethics Committee.

### Photograph processing

Nest material choice in wild birds has typically been assessed by quantifying the materials present in a nest after the young have fledged, when nests can be safely collected and deconstructed. Inferences are then made about the behaviors that led to the observed relative quantities and position of materials in the nest (eg Mainwaring and Hartley 2008; Glądalski et al. 2016; Biddle et al. 2017), but building itself cannot be captured. Therefore, nest deconstruction is not suitable for investigating decision-making *during* building. To examine building parameters such as the timing and rate at which the nest-building bird brings materials to the nest, we analyzed images of the upper nest surfaces taken every 2 d throughout nest building. Note that a photograph of a surface cannot indicate the depth of any materials present and so we cannot infer the composition of the rest of the nest (Sugasawa et al. 2021).

We analyzed 767 nest photographs taken of 62 nests built by 31 female blue tits in 2017 (n_nests_ = 13), 2018 (n_nests_ = 22), 2019 (n_nests_ = 9), 2021 (n_nests_ = 9) and 2022 (n_nests_ = 9), ie two nests per bird. We selected nests for analysis if they were built by a ringed (individually identifiable) female who built a nest in two consecutive years, when she was aged 1 and 2 yr old, and only if she completed both nests, ie at least one egg laid in each year. We analyzed all photographs of selected nests. We note that the nest observation schedule meant that birds could begin to add material between checks and therefore before the first photograph (for up to 4 d for the first bird to add material that season, and for up to 2 d for all the subsequent females that commence building that season).

**Fig. 1.**
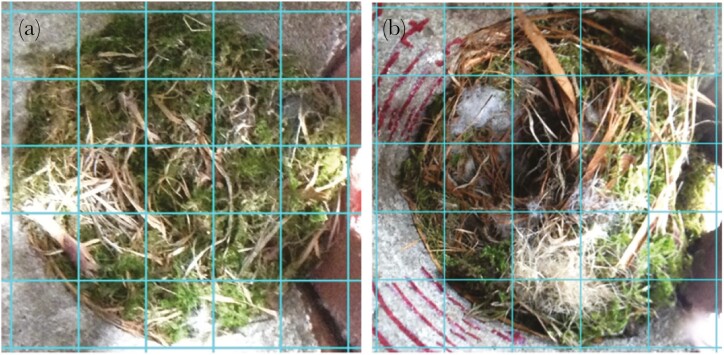
Examples of nest surface photos with 5 × 5 grids overlaid. A) Nestbox J006, on 2018.04.23. A circular nest comprised mainly of structural grass and moss. B) Nestbox J012, on 2019.04.07. An oval nest, with only three sides of the surface touching the edges of the grid (the left side is not touching the grid edge). Insulation addition is underway, with several clumps of hair visible in the photo.

We estimated the relative material composition of the upper surface of the nest as percentages of each material type using ImageJ (Schneider et al. 2012). We first rotated all photos so that the entrance to the nestbox was on the right-hand side of the image, then overlaid a 5 × 5 grid on the photo (Grant et al. 2021) so that the circular border of the nest surface touched each outer border of the grid (Fig. 1A). Most nest surfaces were circular, due to the cylindrical inner chamber of the nestbox, but where they were oval (due to the angle at which some of the photographs were taken), we ensured the grid contained the whole nest surface by aligning three outer borders of the grid with three edges of the nest, and ensured the remaining nest edge was within the grid (Fig. 1B). The size of the grid therefore varied according to the size of the photo. For each cell in the grid, we recorded which materials were present, categorized as moss, hair, grass/leaf (grass from here onwards), feathers, other (eg nestbox interior, iButton, bird, egg), man-made (artificial nest material such as plastic or colored string), or unknown (due to poor lighting or image quality). Since there were 25 cells, each cell contributed 4% of the total surface composition. We calculated the percentage composition of each material by assuming that material types were present in a cell in equal quantities. A cell containing only one type of material contributed four percentage points towards the sum of that material’s total. Cells containing multiple types of material were split equally depending on the number of material types identified in that cell. For example, four types of material in a cell meant that each was assigned one percentage point towards the sum of its total.

Finally, we summed the percentage cover values for feather and hair, and separately the values for grass and moss, to give the proportions of the nest surface in each photo covered by insulation material and structural material respectively (see Fig. S1, Supplementary Material, for an example). As blue tits vary the insulating materials used to construct the nest in response to availability (Britt and Deeming 2011), by summing the values for materials based on function, we aimed to control for differences in abundance of different material types near to each nest site.

### Statistical analyses

The number of fledglings produced from all 204 nests in our study sample followed a zero-inflated bimodal distribution (with mean: 3.5, standard deviation: 3.1, range: 0 to 9). We used a log survivorship analysis from the R package *segmented* to identify two breakpoints in this distribution (Muggeo 2008), at one and seven fledglings. We used these thresholds to define levels of reproductive experience based on the number of fledglings each female produced from her previous year’s nest (no fledglings, between one and six fledglings, or seven or more fledglings). If the female was a yearling with no previous nest, we denoted her reproductive experience “First-Time.” We excluded nests if we could not confirm the female’s reproductive experience in that year (due to missing records of her reproductive history as a result of her choosing not to use our nestboxes in some years), or if it was the second nest built by a bird in the same year (an uncommon occurrence, usually resulting from the failure of the first nest).

To confirm that our study population of blue tits follow the expected pattern of increasing breeding success with experience, we fitted a Generalized Linear Mixed Model (GLMM) with a poisson error distribution and log link function using *glmmTMB* (Brooks et al. 2017). The response variable was number of fledglings from the current nest. The explanatory variables were experience level (First-Time/0 fledglings/1 to 6 fledglings/more than 6 fledglings), and year, with a random effect of mother identity to account for pseudoreplication as a result of having multiple nests per female, and to control for female quality. To account for the high number of nests with no fledglings, we included a zero-inflation component (with a logit link, constraining the response between 0 and 1) and allowed it to vary by experience level and year (Table S1A, Supplementary Material). This allowed us to model both the probability of producing at least one fledgling, as well as the total number of fledglings. We analyzed all nests for which we could determine reproductive experience in the previous year or classify them as First-Time (n = 182), leaving us with 83 First-Time nests, 18 nests where the female had not produced any fledglings the previous year, 52 nests where the female had produced between one and six fledglings the previous year, and 29 nests where the female had produced more than six fledglings the previous year.

Next, to investigate whether nest-surface materials varied between experience levels and in relation to temperature, we fitted two GLMMs with beta error distributions and logit link functions using *glmmTMB* (Brooks et al. 2017). Ecological literature varies in the statistical approaches taken to analyze non-count derived proportional data, with alternatives including a binomial distribution with zeros removed by adding a very small value to them, arcsine transformation, and logit transformation. As in our data, where the relationship is suspected to be non-linear, or where heteroscedasticity exists, it is preferable to model proportional data with a beta distribution without transforming them, to minimize bias in the response variable (Ferrari and Cribari-Neto 2004; Cribari-Neto and Zeileis 2010; Douma and Weedon 2019).

For both materials models, we used the subset of nests (n = 62) for which we had processed photographs (n = 767) in a paired study of the same female birds (n = 31) over two consecutive years. There were 31 First-Time nests, 10 nests where the female had not produced any fledglings the previous year, 12 nests where the female had produced between one and six fledglings the previous year, and 9 nests where the female had produced more than six fledglings the previous year.

The random effects were clutch identity (the unique identification number of the nestbox plus the year, to account for pseudoreplication arising from having multiple photos of each nest, and to control for possible effects of territory and mate quality) and mother identity (to account for pseudoreplication arising from having two nests per female, and to control for female quality). Nineteen nests had autocorrelated consecutive observations of both structural and insulation cover, therefore we fitted a clutch identity-specific autoregression term in each model to correct for the influence of the preceding proportion of material cover on the next observation.

For both material types, exploratory plots suggested greater variation in cover as building progressed. Therefore, in each model we allowed the dispersion (with a log link, to constrain the response to positive values) to vary according to experience level and nest building-progression.

For insulation material cover, the response variable was the proportion of the nest-surface cover that was insulating material (Table S1B, Supplementary Material). The independent variables were year (to control for differences in food availability and long-term temperature trends), building progression (the number of days into nest building on which the photo was taken, divided by nest-building duration in days (mean: 23.4, standard deviation: 9.3, range: 8 to 46), to give the proportion of nest-building duration that had elapsed on the day of the photo) and its square (because exploratory plots suggested a non-linear relationship with the response variable), number of fledglings from the current nest, previous reproductive experience (with four levels as in the model for reproductive success), and the mean temperature across each of the three, seven, or 14 d prior to the date on which the photo was taken (in separate models). Temperature and number of fledglings were scaled to aid interpretation of effect sizes. Since we expected variation in the rate of nest building due to number of fledglings (our first hypothesis that reproductive success is associated with earlier or faster inclusion of insulation material) and experience level (our second hypothesis that repeatability of nest building behavior depends on prior experience of success), we included 2- and 3-way interaction terms between experience level, number of fledglings and building progression. To test our third hypothesis that response to temperature depends on prior experience of success, we included a final interaction term between experience level and mean temperature.

As blue tits tend to start nest-building with structural material (Mainwaring and Hartley 2008), we expected there to be more zero values for insulation cover at the start of nest building. We therefore allowed the zero-inflation of the model (with a logit link, to constrain the response between 0 and 1) to vary by experience level and building-progress proportion. The zero-inflation model describes the probability of obtaining an extra zero value for insulation cover, and its complement, the probability of obtaining a non-zero value, becomes a multiplier to the fixed effects model to generate the linear predictor.

For structural material cover, the response variable was the proportion of the nest-surface cover that was structural material (Table S1C, Supplementary Material). The independent variables and their interactions were the same as for the insulation material model, except that building progress was cubed instead of squared (indicated by exploration of the observed values and confirmed by comparing goodness of fit plots for models containing either a quadratic term or a cubic term for build progress).

We completed data analysis in R v.4.2.2 (R Core Team 2022). We calculated P values for mixed models using *lmerTest* (Kuznetsova et al. 2017). We assessed significance of terms in the fitted models using Type II Wald chi-square tests from *car* (Fox and Weisberg 2019). Post hoc tests for differences between levels of independent variables used Tukey contrasts generated by *multcomp* (Hothorn et al. 2008) and *emmeans* (Lenth 2022). We assessed model fit visually using diagnostic plots in *DHARMa* (Hartig 2022).

### Temperature variable selection

Since this is the first study to attempt to analyze which materials are included into the nest during building, it was not clear which time period and what temperatures might influence material selection. However, as Britt and Deeming (2011) found that when temperatures were lower in the 7 d prior to egg laying the total mass of blue tit nests was larger, we used a model-selection process to identify whether the 7-d period prior to each photograph was most likely to influence nest material cover. We also looked at two other periods, one shorter (3 d prior to each photograph) and the other longer (14 d prior to each photograph) and compared the three models using their Corrected Akaike Information Criteria (AICc).

All three models fitted to investigate structural material coverage had similar AICcs (Table S2A, Supplementary Material), suggesting little difference in the ability of each temperature variable to predict structural cover. We selected the model using the 7 d prior to each photograph as it had the lowest AICc, and to enable an intuitive comparison with insulation cover. Of the three models fitted to investigate insulation material coverage, the model including the mean temperature over the 7 d prior to the date of the photograph had an AICc at least two lower than both comparison models (Table S2B, Supplementary Material).

## Results

### Reproductive success

**Fig. 2.**
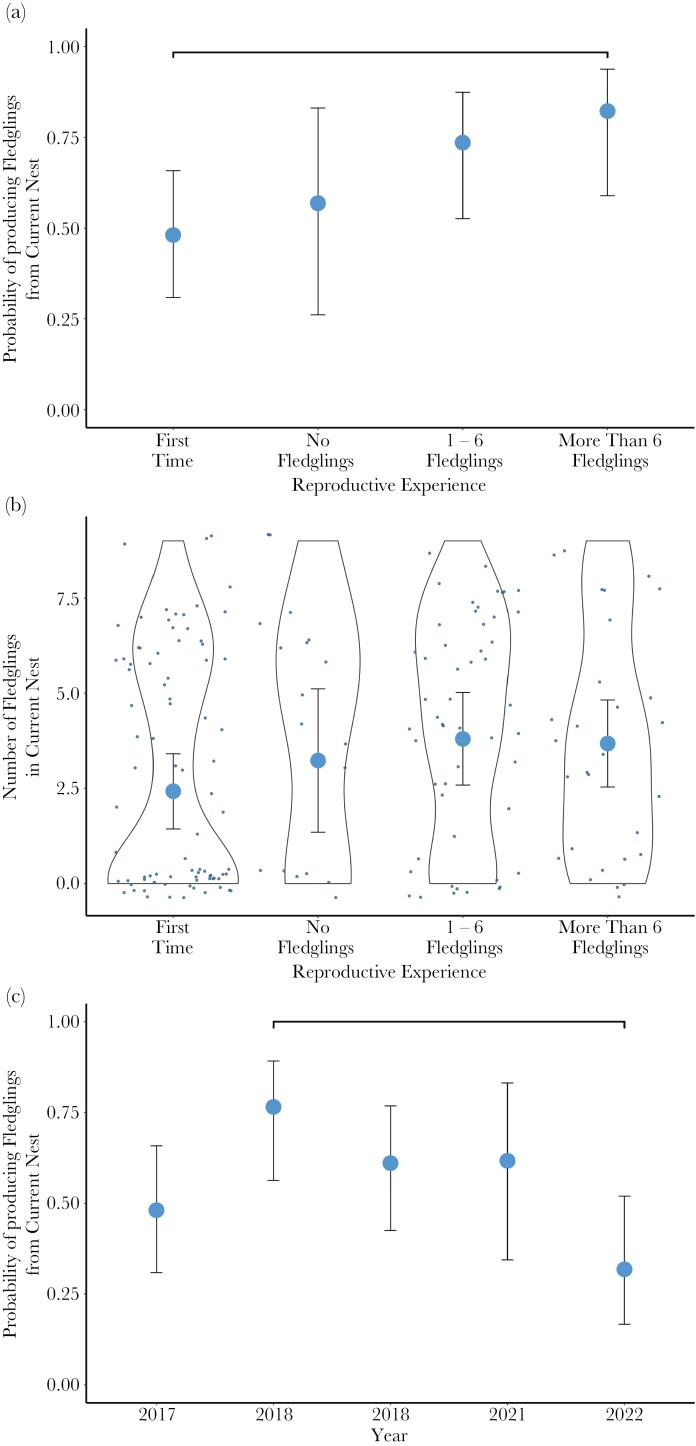
Estimated reproductive success rates (n_nests_ = 182). A) Mean probability of producing at least one fledgling by reproductive experience, corrected for year (*: *P* = 0.04). B) Mean total number of fledglings produced by experience level corrected for year (large points). Small points show the observed number of fledglings produced, and violin plots show the distribution of number of fledglings across experience levels. C) Mean probability of producing at least one fledgling by year, corrected for experience (**: *P *= 0.01). Error bars represent 95% confidence intervals.

Reproductive experience affected the probability of a bird producing at least one fledgling in its next nest (GLMM^zi^: χ^2^_3_ = 10.84, *P* = 0.01): birds that had previously fledged more than six nestlings were more likely to fledge at least one in their next nest, compared to birds breeding for the first time (odds ratio = 5.0, S.E. = 3.0, *P* = 0.04; Fig. 2A; there was no evidence for a statistical difference in probability across any other pairwise combinations of experience level). However, previous successful experience did not result in a greater total number of fledglings (GLMM: χ^2^_3_ = 2.31, *P* = 0.51, Fig. 2B). Indeed, birds that had previously produced more than six fledglings tended to produce fewer from their next nest (predicted mean = 4.47 fledglings). Total number of fledglings followed a markedly different distribution depending on experience: First-Time birds usually produced either none or around six fledglings, whereas experienced birds had a more uniform distribution between zero and nine fledglings (Fig. 2B).

The probability of producing at least one fledgling was greatest in 2018 and lowest in 2022 (GLMM^zi^: χ^2^_4_ = 13.19, *P* = 0.01; 2022 compared to 2018: odds ratio = 0.14, S.E. = 0.08, *P* = 0.01; Fig. 2C). However, the total number of fledglings produced did not differ across years (GLMM: χ^2^_4_ = 4.60, *P* = 0.33).

### Insulation material over time

**Fig. 3.**
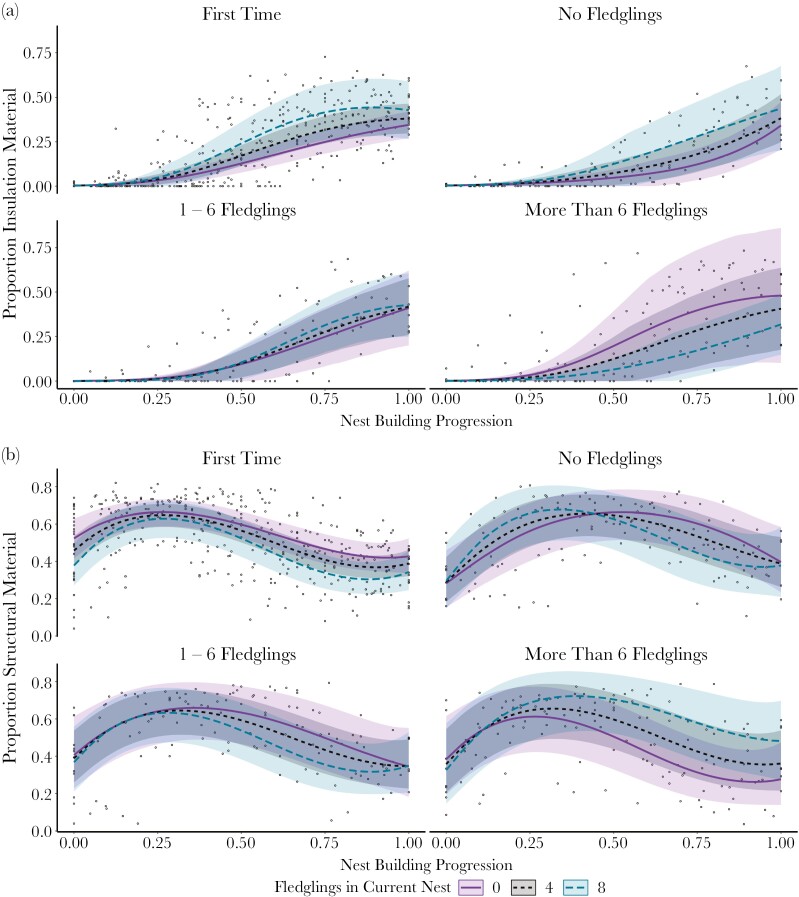
Model predictions by previous reproductive experience for A) insulation cover and B) structural cover. Lines represent the predicted proportion of the nest surface covered in each material as nest building progressed. Points represent observed values of nest-material coverage. Shaded areas represent 95% confidence intervals of the predictions. Number of fledglings produced from the current nest was a continuous variable, so for clarity, predictions are shown for selected numbers of fledglings, with values chosen to represent the middle of each segment of the fledglings distribution (0, 1 to 6, and 7 to 9 fledglings). Purple solid lines = no fledglings produced from current nest; black dotted lines = 4 fledglings produced from current nest; green dashed lines = 8 fledglings produced from current nest.

For all nests, insulation cover started near zero at the beginning of nest building and increased as nest building progressed (GLMM: χ^2^_2_ = 212.19, *P* < 0.001; Fig. 3A). The probability of having any insulation material in the nest surface varied due to experience level (GLMM^zi^: χ^2^_3_ = 10.06, *P* = 0.02): birds that had fledged between one and six fledglings in their previous nest were more likely to have insulation material in their nest surfaces than were first-time birds (odds ratio = 0.42, S.E. = 0.12, *P* = 0.02). Birds that had previously fledged no, or more than six nestlings, had intermediate levels of insulation materials in their nests. However, as expected, the main explanation for a nest containing any insulation material was its progression through nest building (GLMM^zi^: χ^2^_1_ = 199.98, *P* < 0.001): all nests increased in insulation cover as nest building progressed (GLMM: χ^2^_2_ = 212.19, *P* < 0.001; Fig. 3A). However, the rate of increase depended on previous breeding experience (GLMM: χ^2^_6_ = 14.92, *P* = 0.02; Fig. 3A): birds that had not fledged nestlings from their previous nest had an initial gradual increase followed by a steep increase in insulation cover later in nest building, whereas first-time birds and those that had raised fledglings previously included insulation early and more rapidly as they built their nests (estimates for the trend in the curve describing the effect of nest building progression on insulation material: first-time = −3.15, S.E. = 0.76, 1 to 6 fledglings = −2.89, S.E. = 1.65, more than 6 fledglings = −2.28, S.E. = 1.65, no fledglings = 1.60, S.E. = 1.43; Fig. 3A).

Nests that produced more fledglings tended to have more insulation material in their surface, except for nests built by birds that had previously fledged more than six nestlings, where the opposite was true (GLMM: χ^2^_3_ = 11.62, *P* = 0.01; Fig. 3A). There was no significant interaction between experience, fledglings and nest building progression (GLMM: χ^2^_6_ = 3.34, *P* = 0.77). There was no evidence for a difference in insulation cover across years (GLMM: χ^2^_4_ = 4.16, *P* = 0.39) and no effect of temperature (GLMM: χ^2^_1_ = 1.17, *P* = 0.28).

The variation between individual nest builders was relatively unimportant in determining insulation cover during nest building as the variance in the mother identity random effect approached zero. There was considerable variance attributed to the nested Clutch ID random effect (var = 0.50), indicating consistent variation within nests which may be due to some other factor we did not measure.

### Structural material over time

All nests had approximately 50% of their surface covered in structural material in their first photograph regardless of previous experience (GLMM: χ^2^_3_ = 0.14, *P* = 0.99; Fig. 3B) or year (GLMM: χ^2^_4_ = 2.32, *P* = 0.68). This, perhaps counter-intuitive, level of coverage is due to the nest observation schedule, which meant that birds had begun nest building before the first photograph was taken. All nests showed an initial increase in structural cover followed by a gradual decline as nest building progressed (GLMM: χ^2^_3_ = 199.79, *P* < 0.001; Fig. 3B). This decline is because once nest material covers the whole surface of the nestbox floor, as insulation cover increases, structural cover must decrease, so the relative proportions of each are complementary in the later stages of building (but not at the beginning of measurement, when a large proportion of the grid contained the nestbox floor, labeled “other material” in our photograph-processing step). The rate of change in structural material cover depended on the reproductive experience of the female (GLMM: χ^2^_9_ = 19.72, *P* = 0.02): birds that had previously not produced fledglings had a higher peak coverage of structural material compared to first-time nest builders (contrast = 2.77, S.E. = 0.92, *P* = 0.01; Fig. 3B).

More fledglings were associated with more structural material only for birds that had previously fledged more than six nestlings (GLMM: χ^2^_3_ = 7.95, *P* = 0.05; Fig. 3B). For the birds with other reproductive experiences more fledglings were associated with less material.

There was no effect of temperature on structural material cover (GLMM: χ^2^_1_ = 0.11, *P* = 0.74) and no interactions between experience, fledglings and nest building progression (GLMM: χ^2^_9_ = 7.42, *P* = 0.59). The variance in each random effect approached zero, suggesting almost no effect of mother identity or nest site on structural cover.

## Discussion

As expected, insulating material cover increased as nest building progressed and egg-laying approached, whereas structural cover peaked partway through building. However, first-time and previously successful birds rapidly increased their nests’ insulation cover earlier during nest building than did previously unsuccessful birds. Nests built by birds that had previously experienced total failure instead had a higher peak in structural material coverage than other nests. The more fledglings produced from a nest, the more insulation in a nest surface there was throughout nest building, except for nests built by birds that had previously raised more than six fledglings. Unexpectedly, we found no effect of temperature on the rate at which the birds brought insulation or structural material.

Our results support previous work across multiple taxa that showed breeding success increases with age and/or breeding attempts (Angelier et al. 2007; Buston and Elith 2011; Dugdale et al. 2011) but suggest that, at least for wild blue tits, their previous breeding *outcome* is also important. First-time nest builders that added insulation sooner and at a higher rate were also more likely to produce young and to produce more young. The successful first timers went on to include insulation in a similar way the following year. These data support the experimental data from zebra finches that successful birds stick with their building behavior second time around. The exception was those birds that had produced at least six young. These birds varied in the amount of insulation visible in their next nest and those that added less than they had the previous year were more likely to produce fledglings. It seems probable that other factors explain their success in spite of reducing the amount of insulation material, which might include changes in behavior that occur after the eggs have hatched, such as an enhanced ability to feed young (see comments below).

Birds that had previously raised no fledglings changed what they did for their second nest, which also echoes in some ways the experimental data from zebra finches. In this case, the previously unsuccessful birds that produced more fledglings had added more insulation sooner into their second nest, whereas those previously unsuccessful birds that continued to be unlikely to be successful continued to be late to begin adding insulation material. Experience does then affect nest-building decisions in wild blue tits, and those decisions depend on that experience.

At least at the relatively northerly latitude of our site, a minimum quantity of insulation material in the nest, achieved by starting to add it well in advance of egg laying, seems necessary for successful fledging. This finding echoes an earlier study that found an increased likelihood for blue tit fledglings to be recruited into the breeding population when the proportion of feathers in the nest was greater (Järvinen and Brommer 2020). However, since nest photos can only show the relative quantities of materials present on the surface of the nest, they do not necessarily reflect the composition of the whole nest when it is completed (Sugasawa et al. 2021). Whether the quicker and earlier addition of insulation material results in greater mass of insulation material present in the whole nest, or greater insulation capability of the nest, requires confirmation from nest deconstruction and calculation of cooling rates (Chapman et al., in preparation).

Why some birds bring insulation sooner than do others is not clear, but the energetic costs of nest building may play a role. Blue tits fed a supplemented diet built heavier nests than did birds not provisioned with food, but because they all took a similar time to complete their nest, the supplemented group must have brought more nest material at a faster rate than did the birds without extra food (Mainwaring and Hartley 2009). Nest building is a time-consuming activity that reduces available time for foraging (Mainwaring and Hartley 2013) and given that reproductive success is a measure of individual fitness (Grafen 1988), some birds may enter the breeding season with low energy reserves, unable or unwilling to build as fast at the beginning of nest building as high-quality individuals.

Based on the experimental work in zebra finches we had expected our first year birds to use temperature as a cue to bring insulation material to their nest. We did not see an effect of temperature on the rate at which the birds brought either category of nest material. Whether that is because the variation in temperature within and across days during nest building is not a useful predictor of the temperature likely to be experienced during incubation or because the birds are more likely to begin building sooner if the temperatures are generally higher is not yet clear. Collecting more data on nest building in this and other species would be helpful in addressing these possibilities.

It is pertinent to note that the production of live fledglings is not just due to the quality of the nest. Other factors such as hours spent incubating, foraging efficiency and offspring provisioning (Kluen et al. 2011), which themselves improve with age or experience in some species (Daunt et al. 2007; Limmer and Becker 2009; Hassrick et al. 2013) will also contribute. In species with altricial young, such as blue tits, the ability of *both* parents to provision their young is essential to survival and fledging: it seems likely that the role of the father in enabling more young to fledge is going to be especially important.

In conclusion, experience of the previous reproductive event shapes the decision making of wild blue tits building their next nest. That decision may or may not lead to similar or better success, however. One of the next questions to address is how birds know or learn about material function so that they choose structural and insulation materials appropriately (Healy et al. 2023).

## Supplementary Material

araf009_suppl_Supplementary_Figure_S1_Tables_S1-S2

## Data Availability

Analyses reported in this article can be reproduced using the data provided by Alexander et al. (2025).
