# Supplementary material for: Previous reproductive success informs nest-building decisions
Source: Behav Ecol. 2025 Feb 6;36(2):araf009. doi: 10.1093/beheco/araf009 (PMC11840717; doi:10.1093/beheco/araf009)
Supplement: araf009_suppl_Supplementary_Figure_S1_Tables_S1-S2 [file araf009_suppl_supplementary_figure_s1_tables_s1-s2.docx]

**Supplementary Material**


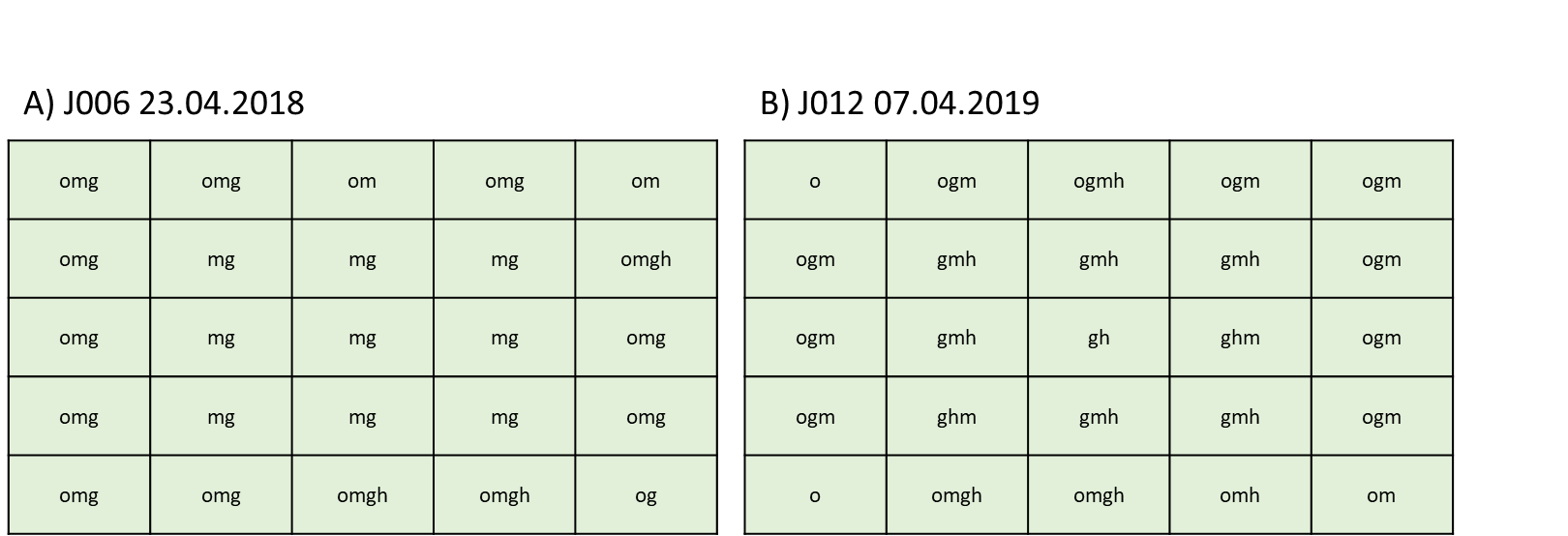


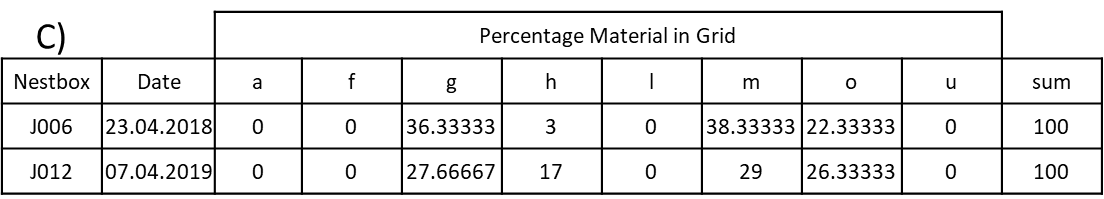


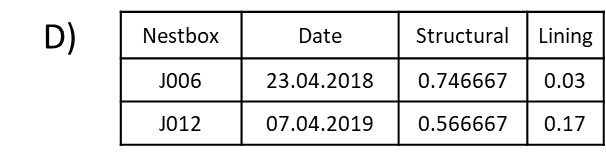


**Figure S1.** Materials found in each square of the 5x5 grid overlaid on photos of A) nestbox J006 on 23 April 2018 and B) nest box J012 on 7 April 2019. These were converted into C) percentages of the total nest surface made up of each material by assuming that each of the 25 grid squares represented 4% of the nest surface area and that when multiple materials were found in one grid square, they were present in equal proportions in that square. A = man-made material; f = feather; g = grass; h = hair; l = leaf; m = moss; o = other; u = unknown. The percentage of “other” material typically falls during nest building as building material progressively covers the nest box floor. Finally, D), the percentages for grass, leaf and moss were added together and divided by 100 to give the proportion (0-1) of structural material in the nest surface, and likewise for feather and hair to give the proportion of lining material.

**Table S1.** Components of regression models. Fledglings = scaled number of fledglings from the current nest; ExpL = reproductive experience; PBD = nest building progression as a proportion of total time taken; mean temperature was calculated across the three, seven or 14 days prior to the photograph date, and were compared in separate models; MotherID = unique identifier for each nest builder, ClutchID = unique identifier for each nest, timeSeries = ordered sequence of photographs for each nest.

| **Response variable** | | **Model Component** | **Formula** |
| --- | --- | --- | --- |
| A) | **Number of fledglings** | Fixed effects | ExpLevel + Year |
|  |  | Zero-inflation | ExpLevel + Year |
|  |  | Random effects | (1\|MotherID) |
| B) | **Lining cover** | Fixed effects and interactions | ExpLevel * (poly(PBD, 2) * Fledglings + $\left\{ \begin{aligned} 3d mean \\ 7d mean \\ 14d mean \end{aligned} \right\}$) + Year |
|  |  | Random effects | (1\|MotherID/ClutchID) +  ar1(timeSeries + 0\|ClutchID) |
|  |  | Zero-inflation | PBD + ExpLevel |
|  |  | Dispersion | PBD + ExpLevel |

| C) | **Structural cover** | Fixed effects and interactions | ExpLevel * (poly(PBD, 2) * Fledglings + $\left\{ \begin{aligned} 3d mean \\ 7d mean \\ 14d mean \end{aligned} \right\}$) + Year |
| --- | --- | --- | --- |
|  |  | Random effects | (1\|MotherD/ClutchID) +  ar1(timeSeries + 0\|ClutchID) |
|  |  | Dispersion | PBD + $\mathrm{ExpLevel}$ |

**Table S2**. AICc for material models using different temperature variables. A difference in AIC greater than 2 typically indicates that a model explains more of the variation in material cover than the comparison models. Bold text indicates the models with the lowest AICc for each material type.

|  | Temperature Variable | AICc | |
| --- | --- | --- | --- |
| A) Structural cover models | Past 3 days | | -1060.27 |
|  | **Past 7 days** | | **-1061.65** |
|  | Past 14 days | | -1059.32 |
| B) Lining cover models | Past 3 days | | -267.42 |
|  | **Past 7 days** | | **-274.64** |
|  | Past 14 days | | -271.82 |
